# Discharge Against Medical Advice From the Emergency Department: Results From a Tertiary Care Hospital in Beirut, Lebanon

**DOI:** 10.1097/MD.0000000000002788

**Published:** 2016-02-12

**Authors:** Mazen El Sayed, Elsy Jabbour, Ali Maatouk, Rana Bachir, Gilbert Abou Dagher

**Affiliations:** From the Department of Emergency Medicine, American University of Beirut Medical Center, Beirut, Lebanon.

## Abstract

Patients who leave the emergency department against medical advice are at high risk for complications. Against medical advice (AMA) discharges are also considered high-risk events potentially leading to malpractice litigation.

Our aim was to characterize patients who leave AMA in a payment prior to service emergency department (ED) model and to identify predictors for return visits to ED after leaving AMA.

We conducted a retrospective review study of charts of ED patients who were discharged AMA between January 1, 2012 and January 1, 2013 at a tertiary care center in Beirut Lebanon. We carried out a descriptive analysis and a bivariate analysis comparing AMA patients without and with return visit within 72 hours. This was followed by a Logistic regression to identify predictors of return visits after leaving AMA.

A total of 1213 ED patients were discharged AMA during the study period. Mean age was 46.9 years (±20.9). There were 654 men (53.9%), 737 married (60.8%). The majority (1059 patients (87.3%)) had an emergency severity index of 3 or less (1 or 2). ED average length of stay was 3.8 hours (±6.8). Self payers accounted for 53.9%. Reasons for leaving AMA were: no reason mentioned (44.1%), incomplete workup (30.5%), refusing admission (12.4%), financial reasons (7.9%), long wait times (2.9%), and others (2.2%). Discharge diagnoses were mainly cardiac (23.4%), gastrointestinal (16.4%), infectious (10.1%), and trauma (9.8%).

One hundred nineteen returned to ED within 72 hours (9.8%). Predictors of returning to ED after leaving AMA were: older age (OR 1.02 95% CI (1.01–1.03)), private insurance status (OR 4.64 95% (CI 2.89–7.47) within network insurance status (OR 7.20 95% CI (3.86–13.44), longer ED length of stay during the first visit (OR 1.03 95% CI (1.01–1.05).

In our setting, the rate of return visit to ED after leaving AMA was 9.8%. Reasons for leaving AMA, high-risk discharge diagnoses and predictors of return visit were identified. Financial status was a strong predictor of return to ED after leaving AMA.

## INTRODUCTION

Against medical advice (AMA) disposition for emergency department (ED) patients is not uncommon yet poorly studied.^1^ Patients usually leave AMA without completing service after ED evaluation and initiation of work-up or after a physician's decision to admit to hospital. Previously reported rates of AMA discharges range from 0.1 to 2.7% of ED visits in UK and US EDs.^2–6^ The American College of Emergency Physicians considers AMA discharges to be high-risk events potentially leading to malpractice litigation.^2,7^ Patients leaving AMA are 10 times more likely to initiate a litigation process against the emergency physician and the hospital than a typical ED patient with a rate of around 1 lawsuit per 300 AMA cases.^3^

Reasons for leaving AMA include, but are not limited to, family obligations, pet care needs, or financial responsibilities.^8^ Dissatisfaction with care provided, long waiting times, and ED crowding are more linked to leaving the ED without being seen by a medical provider rather than leaving AMA.^1,5^

Specific patients’ characteristics are also linked to AMA discharges. Patients leaving AMA are significantly more likely to be young adults, males, uninsured, with low socioeconomic status and to have a lower triage acuity level or a history of previous or current substance abuse.^5,9^ In addition to that, some chief complaints are associated with high AMA visit rates. These include nonspecific chest pain, abdominal pain, headache, nausea, and vomiting.^5^ Factors driving patients with such diagnoses to leave AMA include length of the ED workup, invasive procedures, perceived wait, and associated discomfort.^5^

Studies examining outcomes (return visits, hospitalization, mortality) of patients who leave AMA are often limited by unsatisfactory response rates at follow-up, small sample sizes, or lack of valid comparison group.^5,6,8,10^ However, patients who leave AMA may be severely ill and at risk of experiencing adverse events^2,5,6^. They are also more likely to return to the ED and be emergently hospitalized within a short time after the initial ED visit.^5,6,8,10^

The potential for improvement in ED processes and patients outcomes by reducing AMA rates is also great. In a study by Robinson et al, patients who left AMA made up 24.4% of all ED revisits.^8^

Unlike EDs in the United States, our ED has a payment before service model where collection of charges is done before providing service except for life-threatening and high-acuity cases. Leaving AMA is common, however the associated factors, patients’ characteristics, and outcomes in addition to predictors of return visits are not clear.

The objectives of this study are to describe characteristics and outcomes of patients leaving AMA, to examine reasons behind this decision, and to identify predictors of return visits to the ED after leaving AMA in an effort to reduce the AMA rate and improve patients outcomes.

## METHODS

### Study Setting and Design

Lebanon has over 168 private hospitals (mostly in large cities) and around 10 public hospitals.^11^ The system has a relatively high utilization rate (12% of the population) with an occupancy rate close to 55% and an average length of stay of 4.5 days. Up to 53% of the population lack insurance coverage and receive support from the Ministry of Public Health. The national health expenditure per capita was reported to be $460 in 2006 with around 44% from out of pocket contribution.^11^

The study was conducted in the ED of the American University of Beirut Medical Center, the largest tertiary care center in Beirut Lebanon, with around 49,000 ED patient visits per year. ED triage is done using emergency severity index (ESI) assigning acuity scores to patients.^12^ ESI is a 5-level tool for emergency department triage used to rate patient acuity from level 1 (most urgent) to level 5 (least resource intensive) based on an estimation of resources required.^12^ The ED has a payment prior to service model whereby patients deemed to have a medium or low acuity condition at triage (ESI 3, 4, or 5) are usually financially cleared prior to service delivery. High acuity patients (ESI 1 or 2) are medically stabilized prior to financial clearance.

A retrospective chart review of all patients who were discharged AMA from the ED between January 1, 2012 and January 1, 2013 was done. The study was approved by the AUB Institution Review Board.

### Inclusion/Exclusion Criteria

AMA is a separate category that is assigned electronically to patients by the discharging provider when they leave the ED in our system. A list of all patients discharged AMA during the study period was generated and their charts were included for review (initial visit). All patients who left AMA had a signed AMA form in their chart. Patients who returned to the ED within 72 hours of AMA discharge (return visit) were flagged and reviewed for additional data collection. A list of 1256 patients was identified with 43 patients excluded because of admission to hospital within the same visit (signed AMA for refusing a treatment with proven benefits or additional work-up needed to reach a clear diagnosis), or missing ED charts (ED records consist of paper charting then scanning combined with electronic tracking). A total of 1213 patients were included in the study.

### Data Collection

The data collection was composed of 2 parts: the sociodemographic characteristics (age, sex, marital status, guarantor), clinical and administrative elements (ESI, diagnosis, ED length of stay (LOS), date of admission and date of discharge, cost of care), and hospital characteristics (ED volume) were retrieved from the ED electronic business intelligence software QlikView. QlikView is a data management system that contains comprehensive health and sociodemographic information and is continuously monitored by personnel in the information technology department for validity and reliability.

Other variables including specialty of the ED physicians, time interval between the admission time “triage” and the time a medical doctor examined the patient (door to doctor time), mode of arrival, reason for AMA, and mortality at 30 days were extracted from the patients’ charts.

The AMA form and process used in our ED are consistent with internationally accepted AMA standards of documentation^13^ which include the following: “Signatures of physician and patient, date and time of AMA, medical condition, risks and benefits, consequences of leaving AMA, mental capacity assessment, understanding of proposed treatment, understanding of consequences of refusing treatment, reason for refusal, self care and when to seek medical attention and further follow-up arrangements.”^13^

### Statistical Methods

Statistical analyses were performed using SPSS 22 (Statistical Package for Social Sciences). In the univariate analysis, descriptive statistics were calculated for the total study sample and the patients who returned back to the ED: categorical variables were summarized by their frequency distributions whereas continuous variables were presented as means, standard deviations, ranges, and percentiles.

In the bivariate analysis, Student *t* test and Pearson *χ*^2^ test were used to assess the significance of the statistical association between the independent variables (continuous and categorical) and the 2 groups (returned to ED after leaving AMA: yes/no). A *P* value of <0.05 was used to indicate statistical significance. The internal consistency (Cronbach's Alpha) was determined to assess the reliability coefficient between 3 research fellows who examined whether the return visit was related to the first visit. Reliability based on Cronbach's Alpha is classified as follows: excellent (Cronbach's Alpha: >0.80), good (Cronbach's Alpha: 080-0.60, moderate (Cronbach's Alpha 0.60–0.40), poor (Cronbach's Alpha <0.40).^14^

A multivariate analysis was performed using logistic regression to find the best model that fits the data and that explains the association between the 2 groups of the outcomes (returned to ED after leaving AMA: yes/no) and all predictor variables. A backward selection procedure was conducted by fitting return visit with all risk factors found to be significant in the bivariate level in addition to those considered as being clinically meaningful. Furthermore, to determine the final best model at discriminating between the categories of the outcome variable (returned after leaving AMA: yes/no), we drew a receiver operating characteristic (ROC) curve. The ROC curve indicated that the final model was good enough at discriminating between the 2 categories of the outcome variable “return visit” with an area under the curve of 0.737 (*P* < 0.001, CI: 0.691–0.783).

## RESULTS

A total of 1213 ED patients were discharged AMA during the study period (Table 1 ). Their mean age was 46.9 years (±20.9) with over half of male sex (53.9%). The majority had received an ESI of 3 or lower (1or 2) at triage (1059, 87.3%). More than half (53.9%) were self-paying patients in an ED where over 60% of patients are insured. Several reasons for leaving AMA were elicited including in descending frequencies; no reason mentioned (44.1%), incomplete workup (30.5%), refusing admission (12.4%), financial reasons (7.9%), long wait times (2.9%), and others (2.2%).

**TABLE 1:**
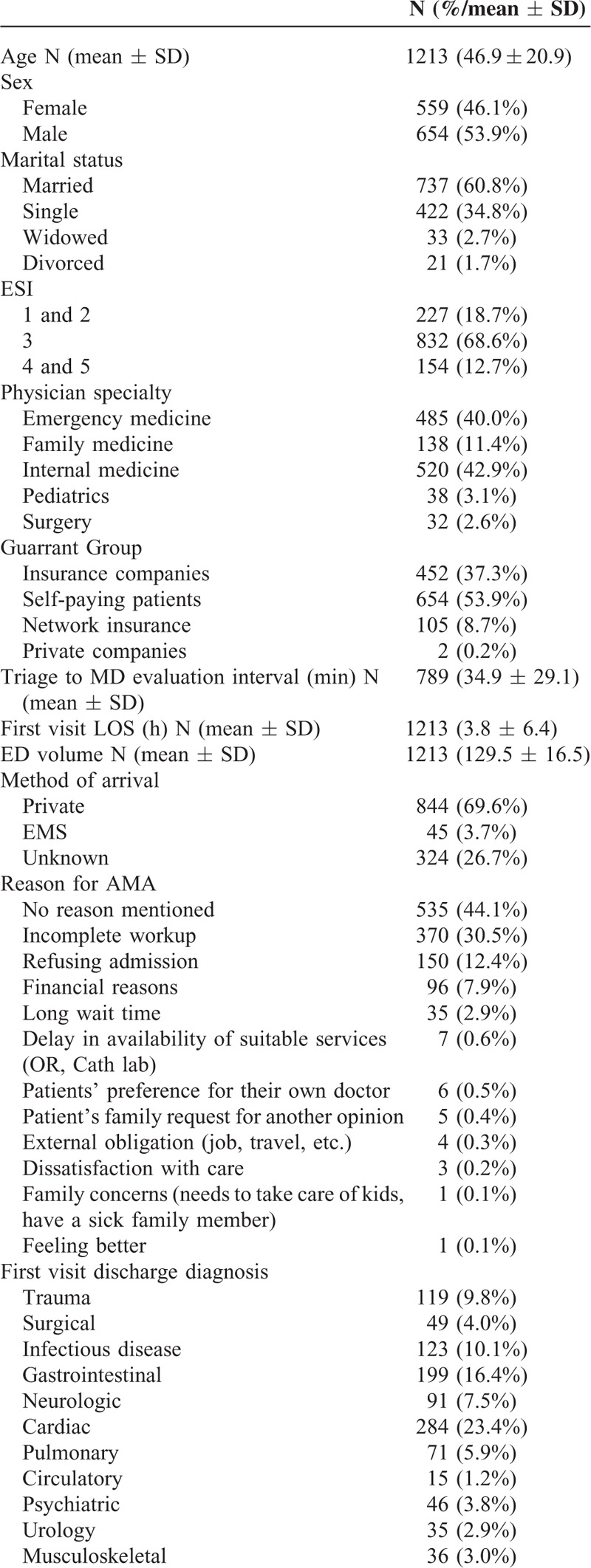
Population Characteristics

**TABLE 1 (Continued):**
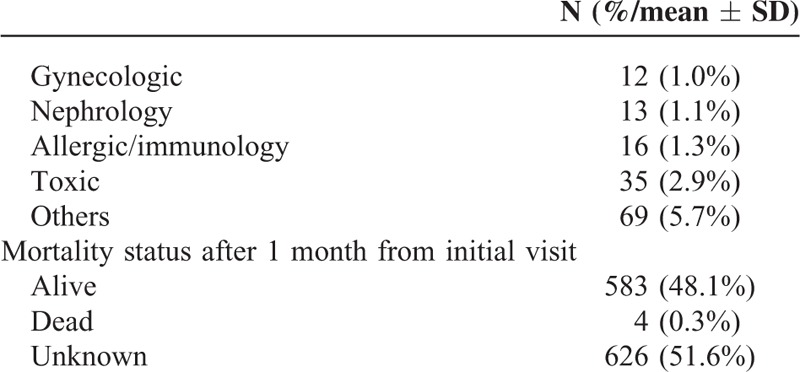
Population Characteristics

Discharge diagnoses were mainly cardiac (23.4%), gastrointestinal (16.4%), infectious (10.1%), and trauma (9.8%). The average ED length of stay during their visit was 3.8 hours (±6.8). At 1-month follow-up, there were 4 documented deaths (0.3%) among those who left AMA.

One hundred nineteen returned to ED within 72 hours (9.8%). Their return visit was directly related to their initial visit as assessed by the research fellow with excellent reliability level and internal consistency (Cronbach's Alpha of 0.935). When compared with those who left AMA and did not return, they were more likely to be older, married, and insured (*P* < 0.05) (Table 2). All of the other variables including ESI, sex, and discharge diagnoses were not found to be significant. The ED daily volumes, ED LOS, and cost of care per ED visit were comparable between the 2 groups (patients without return visit vs. those with return visit).

**TABLE 2:**
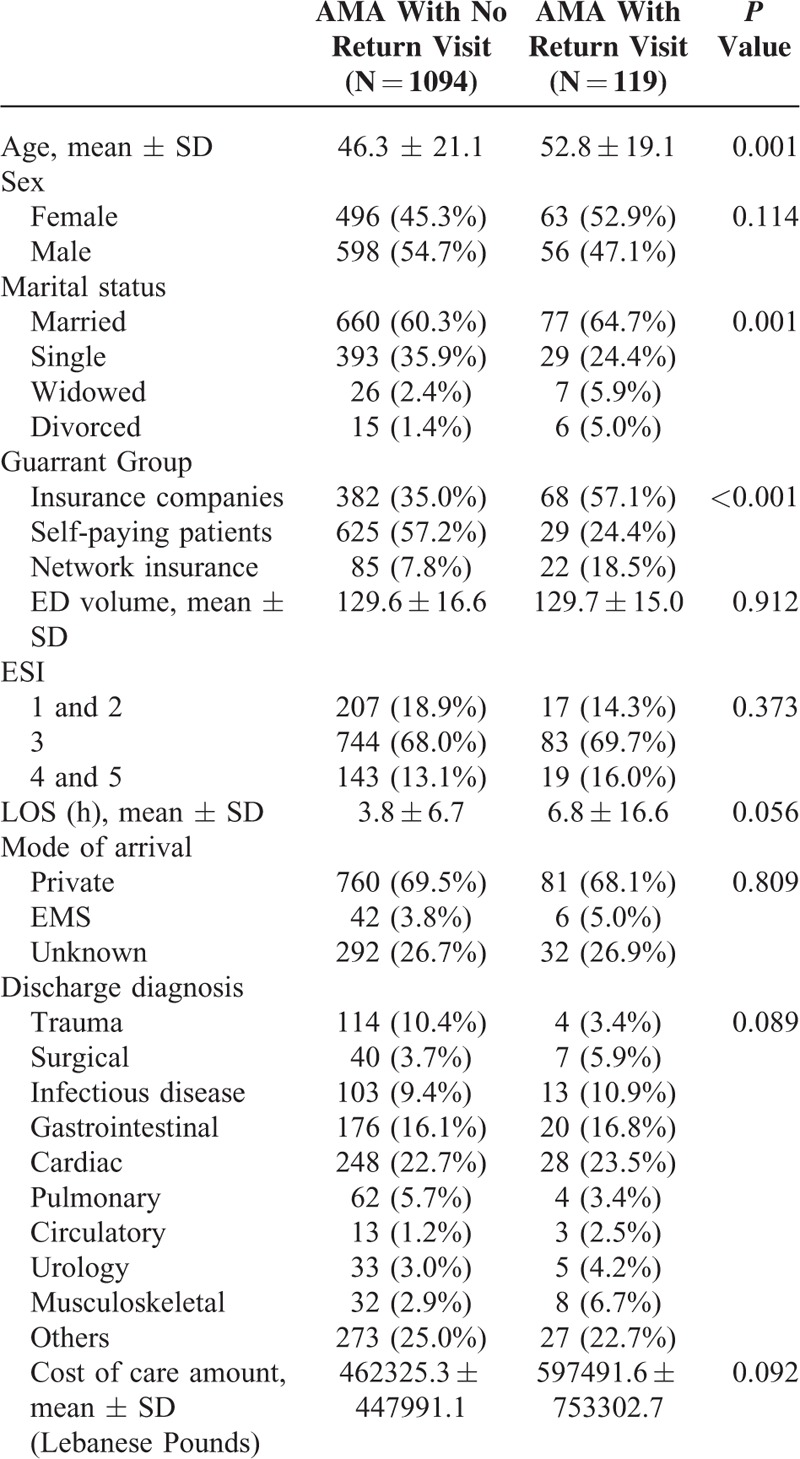
Comparison of 2 Groups of AMA Patients: Those Without Return Visit Vs. Those With Return Visit

Significant predictors of return to ED within 72 hours of leaving AMA included (Table 3): older age (OR 1.02 95% CI (1.01–1.03)), private insurance status (OR 4.64 95% (CI 2.89–7.47)) within network insurance status (OR 7.20 95% CI (3.86–13.44)), longer ED LOS during the first visit (OR 1.03 95% CI (1.01–1.05)).

**TABLE 3:**
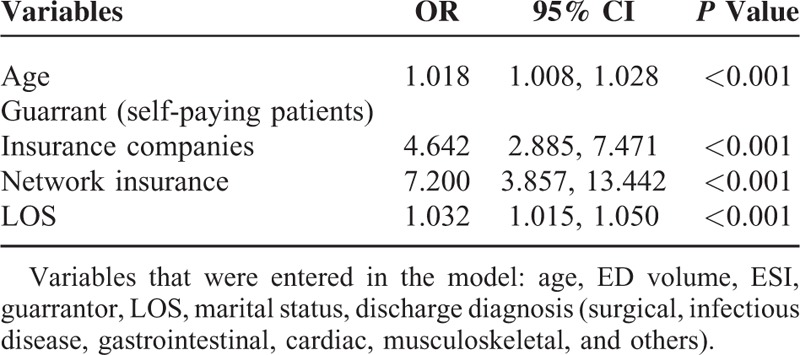
Predictors of Return Visits After Leaving AMA

## DISCUSSION

AMA discharges generate important challenges to ED physicians who need to balance the respect of patients’ autonomy and the necessity for complete evaluation and care. Patients who leave AMA require increased care resources because they often return with worsening clinical status.^5^ A good knowledge of reasons for leaving AMA, of the ethical and legal issues involved can improve the approach to such population.^15,16^

Our study examined characteristics of patients and reasons for leaving AMA from an ED with payment prior to service model in a tertiary care center and identified predictors of return visit in a country where access to care is not well described. The rate of return post AMA was 9.8% (119 patients), which was higher than rates reported in previous studies.^2,6,17–19^ Similar to other settings, patients who left AMA were relatively young (mean age 46.9 years) and uninsured. A study by Ding et al^5^ in a urban inner US city teaching hospital found that patients who left AMA were significantly more likely to be uninsured or covered by Medicaid (US public funded insurance) compared with those who were admitted or discharged. The mean age in their population was 41 years.

Specific chief complaints and diagnoses mainly those that are gastrointestinal or cardiac related accounted for the majority of cases that left AMA. These diagnoses, previously identified by other studies to be linked to AMA,^5^ are usually associated with more prolonged and extensive ED workup including imaging studies (computed tomography scans) or repeat cardiac enzymes.

Furthermore, patients who left AMA after the first visit were mainly seen by an emergency medicine specialist (40%) or an internal medicine specialist (42.9%). This reflects more the practice in our setting where patients with moderate to high acuity (ESI 1, 2, and 3) are seen by those 2 specialties rather than a tendency related to a specific specialty or difference in practice between specialties.

Several reasons for leaving AMA were also identified in our population. These included incomplete ED workup, refusal of admission to hospital in addition to financial constraints. These reasons offer ED physicians and administrators an opportunity to reduce the AMA rates by employing different strategies including improved patient education, shared decision making, acceptable treatment alternatives to both providers and patients, enlisting the help of family members in decision making and a clear explanation about potential adverse events resulting from leaving without completing treatment. Several practices were actually changed in our ED since this study to reduce the rates of AMA. These consist of involving the primary care physician of the patient and the ED case manager in the discussion. Our AMA form was modified accordingly to include documentation of notification and discussion with the patient's primary care physician. Other measures that were implemented include helping patients cover part of the charges if needed through charity care, increasing arranged transfers to other less expensive community hospitals, and introducing phone follow-up with patients discharged AMA.

Our study was also the first to examine predictors of returning to ED after leaving AMA. Our goal was to identify cases with higher likelihood of adverse events and potentially prevent the decision of leaving AMA. Financial coverage status was the strongest predictor of return to ED after leaving AMA with an OR 4.64 95% (CI 2.89–7.47) for private insurance and OR 7.20 95% CI (3.86–13.44) for within network insurance. This reflects the access barrier in our setting for patients who are uninsured and who might not return to ED despite experiencing adverse events because of financial constraints. Other predictors such as increased ED stay during the first visit and older age were also identified. The ED LOS is mainly related to the diagnoses of corresponding patients including the associated prolonged workup previously mentioned. Older age is in general more associated with adverse events and not only in patients who leave AMA.^20^ Among those who returned, 64.7% were married which may have influenced their decision to return.

The results of our study should be considered in the light of its limitations. One limitation is inherent to the retrospective nature of the study. Some of the reasons for leaving AMA were not documented in the patients’ charts and were not identified; however, recently we have implemented in our ED an electronic process whereby physicians are prompted to enter the reason for the decision when an AMA disposition is chosen at discharge. This will enable us to better characterize the reasons for leaving AMA. Another limitation is related to follow-up of patients who left AMA. Although a large proportion of our patients lacked follow-up information, more than 50% of our ED patients are usually returning patients who get their care mainly at our hospital. At 1 month follow-up we had only 4 deaths (0.3%) which may be an underestimate related to loss of follow-up. Our study also did not assess the impact of comorbidities as a potential cofounder and how it is related to age or return visit. Additionally, although not assessed by our study, the impact of cost of care on AMA rates may be different in our setting compared with other community hospitals in Lebanon with similar payment model since the costs at tertiary care centers are usually higher.

This study however raises many important issues some of which are applicable to other EDs in general. Our findings are important for other EDs contemplating adopting a payment before service model and can provide essential information for ED administrators and physicians regarding high-risk cases to potentially prevent or minimize adverse outcomes in affected patients. Although interventions should aim at reducing risk by addressing causes of AMA, several steps can be taken to mitigate AMA-related complications. First, physicians need to document patient's capacity to understand the risks associated with the decision. Second, all attempts should be made to prevent an AMA decision by offering the patient all treatment options available. Third, clear documentation of events, reason for AMA, and an informed consent must be included in the chart.^16^ Last but not the least, if all fails, patients should be offered the option to return to ED at anytime if their condition changes or if they change their mind. Additional measures including contacting the patient's primary care physician and arranging for close follow-up are also needed. Additional system-wide measures such as mandating documentation standards for AMA discharges and offering potential solutions mainly to address financial factors leading to AMA decisions are also important considerations for ED administrators to reduce AMA rates.

## CONCLUSION

In our setting, the rate of return visit to ED after leaving AMA was 9.8%. Reasons for leaving AMA, high-risk discharge diagnoses and predictors of return visit were identified. Financial coverage status was a strong predictor of return to ED after leaving AMA. These findings can be used for process and care improvement and as a basis for testing different strategies to reduce AMA rates in similar settings.

## References

[1] Jerrard DA Chasm RM Patients leaving against medical advice (AMA) from the emergency department-disease prevalence and willingness to return. *J Emerg Med* 2011; 41:412–417. 20097503 10.1016/j.jemermed.2009.10.022

[2] Dubow D Propp D Narasimhan K Emergency department discharges against medical advice. *J Emerg Med* 1992; 10:513–516. 1430994 10.1016/0736-4679(92)90289-6

[3] Monico EP Shwatrz I Leaving against medical advice: facing the issue in the emergency department. *J Healthc Risk Manag* 2009; 29:6–9. 19908647 10.1002/jhrm.20009

[4] Henson VL Vickery DS Patient self-discharge from the emergency department. Who is at risk? *Emerg Med J* 2005; 22:499–501. 15983086 10.1136/emj.2003.005447 PMC1726841

[5] Ding R Jung JJ Kirsch TD Uncompleted emergency department care: patients who leave against medical advice. *Acad Emerg Med* 2007; 14:870–876. 17766732 10.1197/j.aem.2007.06.027

[6] Geirsson OP Gunnarsdottir OS Baldursson J Risk of repeat visits, hospitalisation and death after uncompleted and completed visits to the emergency department: a prospective observation study. *Emerg Med J* 2013; 30:662–668. 22983976 10.1136/emermed-2012-201129

[7] Selbst SM Leaving against medical advice. *Pediatr Emerg Care* 1986; 2:266–268. 3797277 10.1097/00006565-198612000-00017

[8] Robinson K Lam B Early emergency department representations. *Emerg Med Australas* 2013; 25:140–146. 23560964 10.1111/1742-6723.12048

[9] Shirani F Jalili M Asl-E-Soleimani H Discharge against medical advice from emergency department: results from a tertiary care hospital in Tehran, Iran. *Eur J Emerg Med* 2010; 17:318–321. 19898241 10.1097/MEJ.0b013e3283334ef1

[10] Lee TH Short LW Brand DA Patients with acute chest pain who leave emergency departments against medical advice: prevalence, clinical characteristics, and natural history. *J Gen Intern Med* 1988; 3:21–24. 3339484 10.1007/BF02595752

[11] Country cooperation strategy for WHO and Lebanon 2010–2015. World Health Organization website. Available at: http://www.who.int/countryfocus/cooperation_strategy/ccs_lbn_en.pdf Accessed December 28, 2015.

[12] Eitel DR Travers DA Rosenau AM The Emergency Severity Index triage algorithm version 2 is reliable and valid. *Acad Emerg Med* 2003; 10:1070–1080. 14525740 10.1111/j.1553-2712.2003.tb00577.x

[13] Levy F The importance of a proper against medical advice (AMA) discharge. *J Emerg Med* 2012; 43:516–520. 21715123 10.1016/j.jemermed.2011.05.030

[14] Portney LG Watkins MP *Foundations of Clinical Research. Applications to Practice*. 2nd ed. Upper Saddle River, NJ: Prentice Hall Health; 2000.

[15] Clark MA Abbott JT Adyanthaya T Ethics Seminars: a best-practice approach to navigating the against-medical-advice discharge. *Acad Emerg Med* 2014; 21:1050–1057. 25269588 10.1111/acem.12461

[16] Levy F Mareiniss DP Iacovelli C The importance of a proper against medical advice (AMA) discharge: how signing out AMA may create significantly liability protection for providers. *J Emerg Med* 2012; 43:516–520. 21715123 10.1016/j.jemermed.2011.05.030

[17] Weingart SN Davis RB Phillips RS Patients discharged against medical advice from a general medicine service. *J Gen Intern Med* 1988; 13:568–571. 9734795 10.1046/j.1525-1497.1998.00169.x PMC1496999

[18] Magauran BG Jr Risk management for the emergency physician: competency and decision-making capacity, informed consent, and refusal of care against medical advice. *Emerg Med Clin North Am* 2009; 27:605–614. 19932395 10.1016/j.emc.2009.08.001

[19] Crilly J Bost N Gleeson H Patients who presented to an Australian emergency department and did not wait or left against medical advice: a prospective cohort follow-up study. *Adv Emerg Nurs J* 2012; 34:357–368. 23111312 10.1097/TME.0b013e3182705efb

[20] Han JH Lindsell CJ Hornung RW Emergency medicine cardiac research and education group internet tracking registry for acute coronary syndromes (i^∗^trACS) investigators. The elder patient with suspected acute coronary syndromes in the emergency department. *Acad Emerg Med* 2007; 14:732–739. 17567963 10.1197/j.aem.2007.04.008

